# Unraveling the Genetic Diversity of Asian Elephants (*Elephas maximus*) in China: Implications for the Conservation of Asian Elephants

**DOI:** 10.1002/ece3.72498

**Published:** 2025-11-14

**Authors:** Xing Yun, Jingshan Wang, Xu Li, Bin Wang, Shaobing Yang, Dusu Wen, Weibin Wang, Ruobing Han

**Affiliations:** ^1^ Key Laboratory of Forest Resources Conservation and Utilization in the Southwest Mountains of China, Ministry of Education/Key Laboratory for Conserving Wildlife With Small Populations in Yunnan/College of Forestry Southwest Forestry University Kunming China; ^2^ Management and Protection Bureau of Yunnan Xishuangbanna National Nature Reserve Xishuangbanna China; ^3^ Management and Protection Bureau of Yunnan Nangunhe National Nature Reserve Nangunhe China

**Keywords:** Asian elephant, biodiversity conservation, genetic diversity, population bottleneck

## Abstract

The Asian elephant ( *Elephas maximus* ), a crucial flagship and umbrella species in forest ecosystems, possesses significant conservation value. However, it has been categorized as endangered due to several factors, including land use change, human disturbance, and climate change. To evaluate the genetic diversity of Chinese Asian elephant populations and provide a scientific basis for the establishment of the Asian Elephant National Park, microsatellite and mitochondrial DNA analyses were conducted on seven populations distributed across Yunnan Province, China. A total of 121 unique genotypes and five mitochondrial DNA (mtDNA) haplotypes were identified. The results revealed that all populations exhibited varying degrees of inbreeding, with the Mengla population showing the highest inbreeding levels. Significant genetic differentiation was observed between the Mengla and other populations. Interestingly, the Yexianggu population exhibits relatively high genetic diversity despite having undergone a population bottleneck and exhibited significant heterozygote excess. Nevertheless, the overall genetic diversity of Chinese Asian elephants remains relatively low compared to that of populations in other countries. To ensure the long‐term viability of the Nangunhe population, it is imperative to facilitate gene flow between this and other populations. Finally, based on geographic subdivision analysis, we propose that the seven Asian elephant populations should be considered as three conservation management units, namely unit 1 (Nangunhe), unit 2 (Mengla), and unit 3 (Yexianggu, Mengman, Puwen, Kongge, and Menga). This study provides a solid reference for the scientific conservation of the Asian elephant in the future.

## Graphical abstract

The seven Asian elephant populations in China exhibit low genetic diversity and varying degrees of inbreeding. Mengla population exhibits the highest level of inbreeding and the most significant genetic differentiation from other Chinese populations. The Yexianggu population has abundant genetic diversity but historically experienced a population bottleneck. The Chinese Asian elephant populations should be divided into three conservation management units: namely unit 1 (Nangunhe), unit 2 (Mengla), and unit 3 (Yexianggu, Mengman, Puwen, Kongge, and Menga).

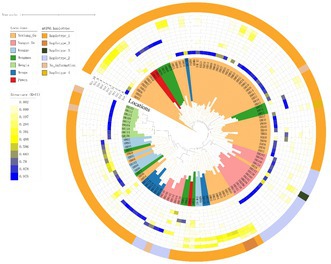

## 1 Introduction

One of the critical steps toward achieving sustainable development is to balance the needs of human development with the protection of the biosphere, on which humans depend (O'Neill et al. 2018). Biodiversity conservation is indispensable for realizing this goal (Opoku 2019), which aligns with the Sustainable Development Goals (SDGs) established by the United Nations. Specifically, SDG 15 emphasizes the commitment to conserving biodiversity and ecosystems. The Asian elephant, a typical herbivore, feeds on a variety of plants by disseminating seeds and facilitating vegetation renewal, thereby playing a vital role in maintaining forest diversity (Campos‐Arceiz and Blake 2011). Additionally, the feces of Asian elephants can promote plant growth and serve as an important source of nutrients in forest soil (Tan et al. 2021). Asian elephants can also create habitable conditions for sympatric species by digging water holes and creating paths, enhancing ecosystem connectivity and improving the adaptability and survival capabilities of these species. This endows Asian elephant conservation with novel scientific significance. It can be inferred that maintaining the evolutionary potential of Asian elephants plays a crucial role in shaping the ecological trajectories of plants, sympatric species, and the entire forest ecosystem (Li et al. 2025; Puri et al. 2019).

The distribution of tropical mammals has decreased by 41% over the past 23 years (1992–2015) due to land use changes and human hunting activities, with large mammals being particularly affected (Gallego‐Zamorano et al. 2020). The same is evident in Asian elephant populations. The Asian elephant has been listed as an endangered species by the International Union for Conservation of Nature (IUCN) since 1986 (Lei et al. 2011). Historically, it was widely distributed across Asia, ranging from West Asia to the Indian subcontinent, along the Iranian coast, and extending to the Yangtze River, covering approximately 9 million square kilometers (Sukumar 2003). In recent years, several factors have contributed to habitat loss of Asian elephants, such as the large‐scale expansion of tropical crops and engineering projects (Wang et al. 2020). Currently, Asian elephants are confined to Asian countries (Vidya 2016), including India, Sri Lanka, Malaysia, Thailand, Myanmar, Indonesia, Bhutan, Laos, Cambodia, Bangladesh, Nepal, Vietnam, and China (Tang et al. 2023). In China, the current distribution range of Asian elephants has shrunk by at least 95% compared to their historical range due to human activities and climatic factors (Bai et al. 2022; Teng et al. 2019; Wan et al. 2019). At present, they are only distributed in southern Yunnan Province (Zhao and Jin 2018). Consequently, it is imperative to develop and implement scientific conservation strategies for Asian elephant populations.

Analyzing population genetic diversity represents one of the critical methods for evaluating conservation efforts, scientific management, and sustainable development for various wildlife species, which also applies to the conservation of Asian elephant populations (Barrett and Schluter 2008; Buckland et al. 2014; Frankham et al. 2011; Saremi et al. 2019). Nowadays, advances in molecular techniques and novel evaluation strategies have facilitated progress in population structure analysis, inbreeding coefficient estimation, and genetic diversity assessment through genome resequencing and single nucleotide polymorphism (SNP) analysis (Forero and Chand 2023). Extensive research on the population structure and genetic diversity of Asian elephants has been conducted over the past two decades to improve the protection of this endangered species (Chen et al. 2022; Fernando et al. 2001; Karuppannan et al. 2019; Kriangwanich et al. 2018; Kusza et al. 2018; Suter et al. 2014; Vidya et al. 2005, 2007). Based on sequences of the cytochrome b gene (CYTB) and the mitochondrial control region (D‐loop), researchers have identified two main clades in Asian elephants population: the α (Indochina‐Laos population) and β clade (Indo‐Myanmar population) (Vidya et al. 2005; Vidya and Sukumar 2005). Both clades have been identified within the Chinese Asian elephant population. Among them, the Nangunhe population belongs to the β clade, while most other populations belong to the α clade (Chen et al. 2022). However, collecting blood and tissue samples from Asian elephants involves a series of complex and varied challenges (Ahlering, Hedges, et al. 2011). mtDNA and microsatellite markers based on fecal sampling provide critical genetic biomarkers for evaluating genetic diversity and delineating management units (Manel et al. 2020; Schmidt et al. 2021, 2022; Stoffel et al. 2018; Theodoridis et al. 2020). Protected area managers and conservation practitioners can use these genomic datasets to strategically design habitat corridor networks and formulate population management protocols, thereby preserving the evolutionary potential of Asian elephants.

Herein, fecal samples were collected from seven Chinese Asian elephant populations. Mitochondrial and microsatellite markers were used to assess genetic diversity and phylogenetic relationships among these populations. Our objectives were to investigate genetic signatures and population bottlenecks across diverse Asian elephant populations, and to compare the genetic variability between the Chinese Asian elephant population and populations in other countries. This information will be crucial for future research on scientific management and breeding within the Asian Elephant Species Survival Plan (SSP). Our results provide a scientific basis for formulating rational conservation strategies for the Asian elephant National Park, and promote biodiversity conservation in the entire forest ecosystems on which their survival depends.

## 2 Materials and Methods

### 2.1 Sampling and DNA Extraction

**FIGURE 1:**
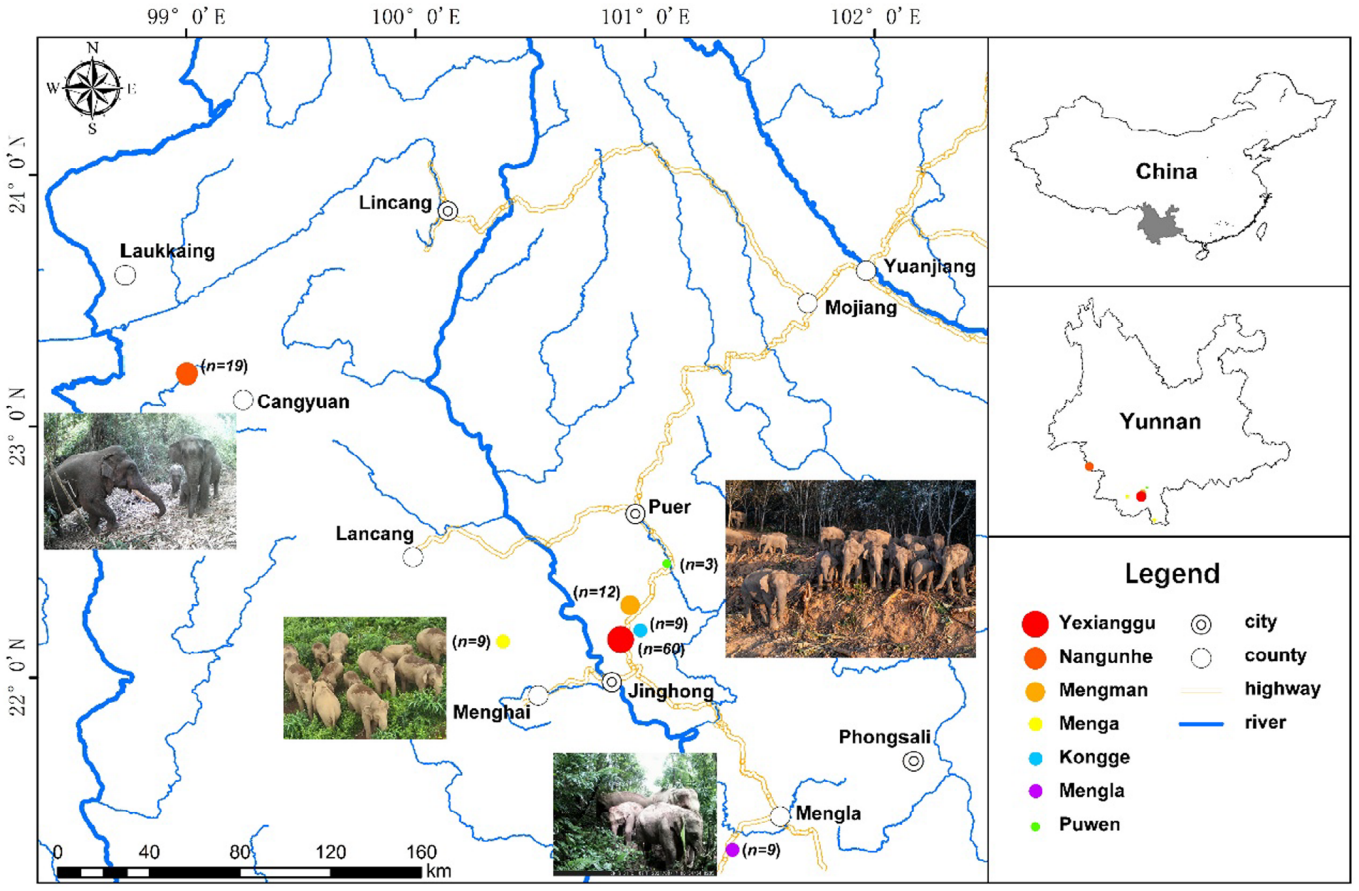
Study area and sampling locations.

From January to May 2023, fecal samples were collected from seven Asian elephant populations in Yunnan Province, China (Figure 1), including Yexianggu, Kongge, Mengla, Menga, Nangunhe, Mengman, and Puwen. After observing Asian elephants defecating, fresh feces were immediately collected with the assistance of forest rangers once the elephants had departed. The samples were placed into 50 mL sterile centrifuge tubes. Disposable gloves were changed after each fecal sample collection. All collected samples were stored at −20°C until DNA extraction. Prior to DNA extraction, fecal samples were vortexed at maximum speed for 3 min, and the mixtures were filtered through a piece of sterile gauze to remove debris. The filtrate was then centrifuged at 9000 rpm for 10 min, and approximately 1 g of the resultant pellet was used for DNA extraction with the QIAamp Stool Mini Kit (Qiagen, Germany). All experimental protocols were reviewed and approved by the Academic Committee of Southwest Forestry University.

### 2.2 Mitochondrial DNA Amplification

The mtDNA, containing the C‐terminal region of cytochrome b (cytb) and the hypervariable left domain of the noncoding control region, was amplified using the previously published primers of MDL3 (5′‐CCCACAATTAATGGGCCCGGAGCG‐3′) and MDL5 (5′‐TTACATGAATTGGCAGCCAACCAG‐3′) (Prithiviraj et al. 2000). The 20 μL reaction system consisted of 2 μL DNA, 10 μL Premix Taq (TaKaRa, DRR003A), 0.4 μM each of forward (labeled 5′‐FAM) and reverse primers, and 1 μg/μL BSA (Sigma). PCR amplification was carried out in a Thermo MBS cycler with initial denaturation at 95°C for 2 min, followed by a touchdown PCR program (45 cycles of 94°C for 15 s, 57°C–58°C for 30 s, 72°C for 45 s), and a final extension at 72°C for 30 min. The amplified products were detected by 1% agarose gel electrophoresis and purified using the Quick Gel Extraction Kit (Watson Biotechnologies, Shanghai, China) (Prithiviraj et al. 2001). The purified PCR products were sequenced on an ABI 310 DNA Sequencer, and the resulting sequences were aligned and examined using BioEdit.

### 2.3 Microsatellite Genotyping

Ten polymorphic microsatellite loci (Table S1) were used to analyze the genetic structure of Asian elephants in Yunnan Province, China (Comstock et al. 2000; Henshaw 2000; Kongrit et al. 2008; Nyakaana et al. 2005). Fecal DNA from all individuals was amplified using a 20 μL reaction system containing 2 μL DNA, 10 μL Premix Taq (TaKaRa, DRR003A), 0.4 μM each of forward (labeled 5′‐FAM) and reverse primers, and 1 μg/μL BSA (Sigma). The PCR protocol consisted of an initial denaturation at 95°C for 2 min, followed by a touchdown PCR program (45 cycles of 94°C for 15 s, annealing temperature for 30 s, and 72°C for 45 s), with a final extension at 72°C for 30 min (Table S1). The annealing temperature was decreased by 1°C every 2 cycles from 56°C to a final touchdown temperature of 47°C, which was used for the following 25 cycles at 50°C. To ensure the results' reliability, each microsatellite locus for each individual was amplified in triplicate. The PCR products were run on an ABI 377 automated DNA sequencer for fragment analysis, and the data were analyzed using GeneScan v3.1.2 and Genotyper 2.5 software (Applied Biosystems). A locus was accepted if it produced the same heterozygous genotype in at least two of the triplicates, and a homozygous genotype was considered if only one allele was detected in all three replicates (Zhan et al. 2007).

### 2.4 Data Analysis

#### 2.4.1 Individual Identification and Genetic Diversity Analysis

MICROCHECKER (v.2.2.3) (Oosterhout et al. 2004) was used to assess potential null alleles and allele dropout (Table S2), and to screen high‐quality data from microsatellite results for subsequent analysis. Based on this, individual identification of all collected Asian elephants was performed using the previously described method to exclude duplicate individuals (Solberg et al. 2006). Genotypes from different samples were considered to be the same individual when all alleles at 10 loci were identical or when a single allele mismatch occurred. The genotypes of microsatellite loci were used to analyze genetic variation by MICROSAT, including the average observed heterozygosity (Ho), average expected heterozygosity (He), average number of alleles per locus, average allele size range per locus, number of unique alleles, and mean variance.

#### 2.4.2 Inbreeding Analysis

The probabilities of full‐sib or unrelated pairs were estimated using GIMLET software (Nathaniel 2002). To investigate parental relatedness among Asian elephant individuals, the triadic maximum likelihood (TrioML) method in Coancestry (Wang 2010) was used to calculate pairwise relatedness value (*R*) and individual inbreeding coefficient (*f*_*M*_) between different individuals based on microsatellite data. Furthermore, the inbreeding coefficient (*F*_IS_) index was statistically validated with 10,000 permutations using molecular variance analysis (AMOVA) in ARLEQUIN 3.1 (Laurent et al. 2005). Specifically, we hypothesize that the seven populations could be categorized into four comparison groups. The first and second groups considered Nangunhe and Mengla populations as independent groups, respectively. The third group consisted of three subgroups, Nangunhe and Mengla as a separate subgroup, and the remaining four populations as another subgroup.

The pairwise genetic relationships between individuals from different populations were assessed through maximum‐likelihood estimation using ML‐RELATE (Kalinowski et al. 2006). The pairwise genetic distances between individuals were calculated in MICROSAT using the *D*_ps_ and *D*_kf_ with the [1—ps/kf] option and 100 bootstraps. These genetic distances were then used to construct a Neighbor‐Joining (NJ) tree using the NEIGHBOR program in PHYLIP (Felsenstein 1989). The NJ phylogenetic tree was visualized using iTOL (Letunic and Bork 2021) and annotated with itol.toolkit (Zhou et al. 2023).

### 2.5 Evolutionary Relationship and Population Differentiation

To investigate the evolutionary relationships among different Asian elephant populations, a phylogenetic tree was constructed in MEGA X using the Minimum Evolution (ME) method (Kumar et al. 2018). The nucleotide diversity (π) of each population was calculated using ARLEQUIN (v3.5) and DNASP (v5.0) (Laurent et al. 2005; Librado and Rozas 2009). Genetic differentiation was assessed using the Fixation Index (*F*_ST_).

### 2.6 Analysis of Genetic Structure

The genetic structure of Asian elephant populations was analyzed using phylogenetic methods and the Bayesian clustering approach implemented in the STRUCTURE software (Jonathan et al. 2000). Specifically, the number of hypothesized *K* values ranged from 1 to 15. The burn‐in and the number of repetitions were set to 50,000 and 10^6^, respectively, with ‘Usepopinfo’ set to 0 under the admixture model. For each value of K, three independent simulations were performed. The highest log‐likelihood value from these iterations was taken as the optimal value, reflecting the most biologically meaningful population structure.

### 2.7 Analysis of Population Bottleneck

To further investigate the genetic diversity among the seven Asian elephant populations, the bottleneck effect was analyzed using the Bottleneck software based on microsatellite data, without prior assumptions regarding historical population structure (Cristescu et al. 2009). The sign test, standardized differences test, and Wilcoxon signed‐rank test (Luikart et al. 1998; Cornuet and Luikart 1996) are commonly used to assess whether a population exhibits a significant excess of heterozygosity. Among them, the standardized differences test is specifically recommended when more than 20 microsatellite loci are analyzed. Therefore, the sign test and Wilcoxon signed‐rank test were applied to evaluate the bottleneck effect in Asian elephant populations.

## 3 Results

### 3.1 Species Identification

After removing duplicate samples, 159 Asian elephants were identified (Figure 1). A total of 121 unique genotypes were identified using 10 microsatellite loci, including 60 in Yexianggu, 9 in Kongge, 9 in Mengla, 9 in Menga, 19 in Nangunhe, 12 in Mengman, and 3 in Puwen. In addition, the population size was estimated to be 155 individuals (95% confidence interval: 136–181) through DNA‐based mark‐recapture analysis using CAPWIRE (Miller et al. 2005).

### 3.2 Haplotype Diversity of Asian Elephant Populations

The mitochondrial cytb sequences from 159 individuals were generated by PCR amplification, and subsequently compared with previously published Asian elephant cytb sequences from the NCBI database, confirming that the collected fresh dung samples originated from Asian elephants.

**FIGURE 2:**
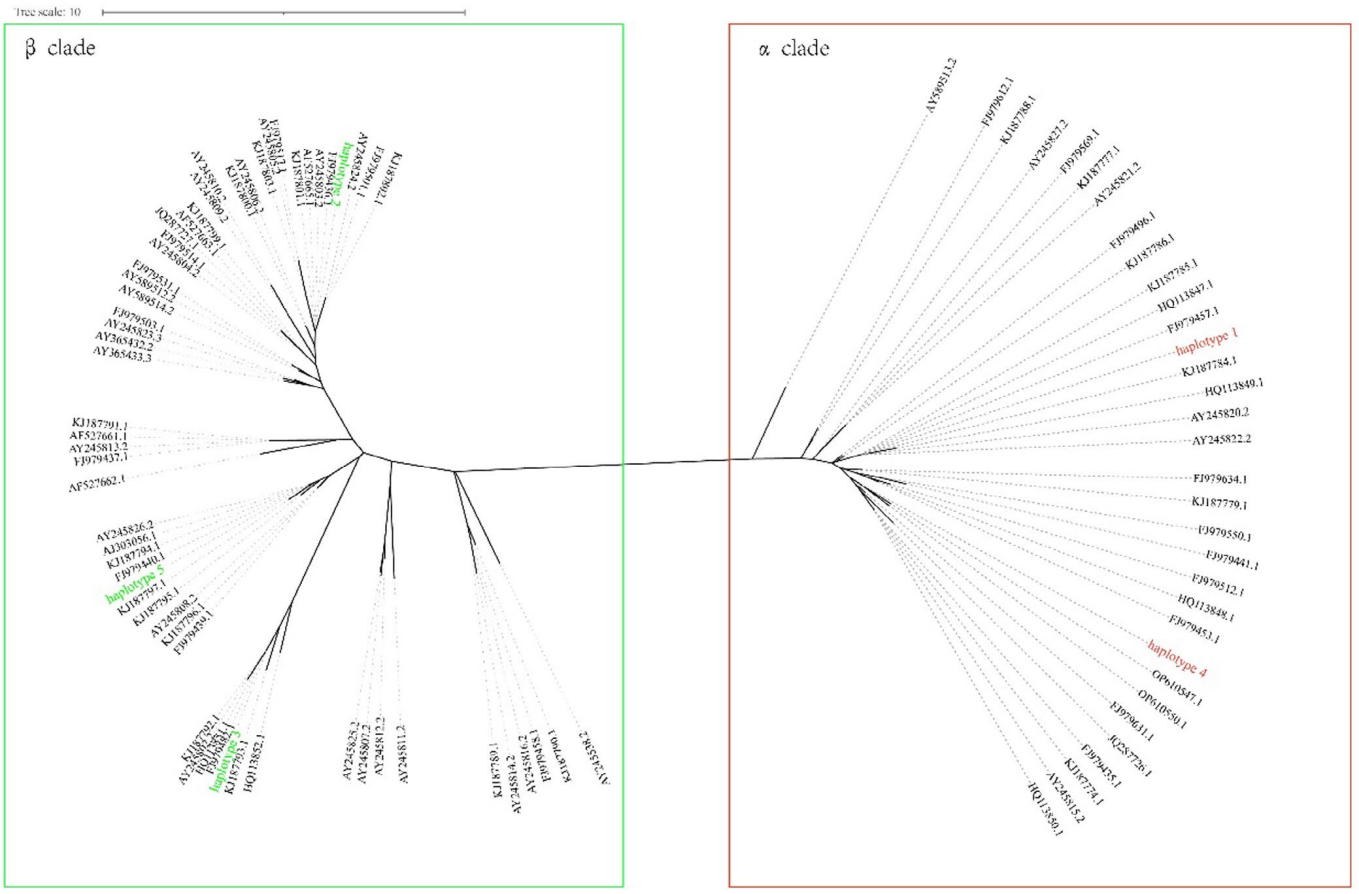
Phylogenetic relationships for Asian elephant inferred from mtDNA sequence. Evolutionary analyses were conducted in MEGA X. The analysis involved 92 nucleotide sequences, and the showed these sequences from this study and other sequences were from NCBI with accession numbers. Sequences belonging to α are highlighted with red rectangular boxes, while sequences belonging to β are highlighted with green rectangular boxes.

**FIGURE 3:**
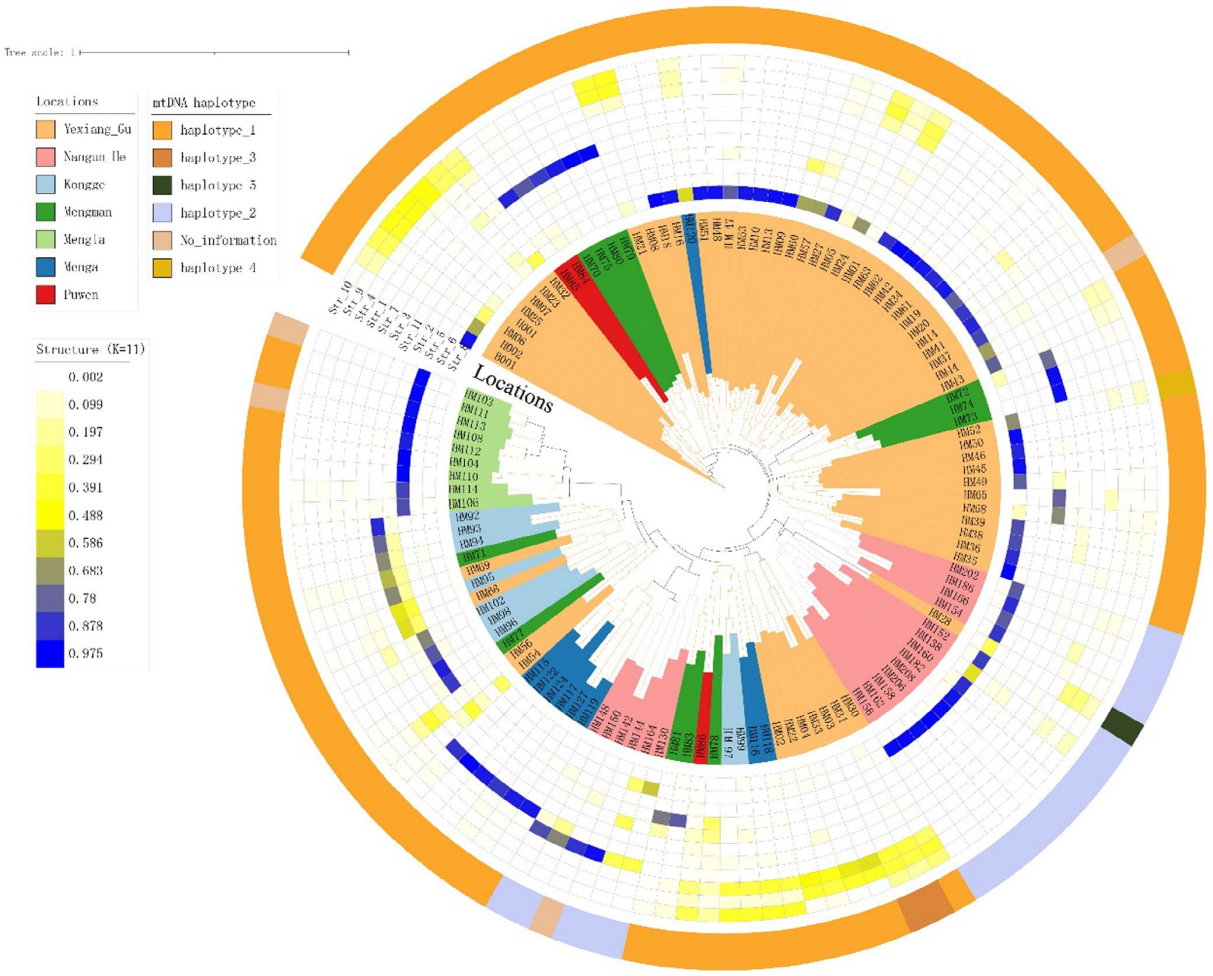
Taxonomic dendrogram of Chinese Asian elephants. Color ranges identify order within the tree. The taxonomic dendrogram was generated with *D*_ps_ distances from the 10 microsatellite loci and displayed with the use of iTOL. The different colors on the phylogenetic tree represent distinct Asian elephant populations. The outer cycle showed the mtDNA haplotypes. The assignment results of individuals following microsatellite genotypes of the 10 loci were shown in the middle cycle. The inner cycle showed the sampled locations.

The length of the mitochondrial cytb sequence obtained was 619 bp. Twenty‐two variable sites resulted in 5 mtDNA haplotypes across the seven Asian elephant populations in Yunnan Province, China. A total of 87 published mitochondrial cytb sequences of Asian elephants were downloaded from the NCBI database, and a phylogenetic tree comprising these 5 haplotypes was constructed using the ME method. The results showed that the 92 cytb sequences could be divided into two distinct clades, namely α and β (Figure 2). The α clade consists of mtDNA haplotypes 1 and 4, while the β clade comprises haplotypes 2, 3, and 5. Haplotype 1, the predominant haplotype in the Chinese Asian elephant population, was distributed across five populations, namely Mengla, Menga, Mengman, Kongge, and Puwen. However, four haplotypes (1, 3, 4, and 5) were identified in the Yexianggu population. Haplotype 2 was exclusively detected in the Nangunhe population (Table 1; Figure 3). The nucleotide diversity (π) of seven Asian elephant populations was calculated. The results showed that only the Yexianggu population showed a π of 1.74050 ± 1.13893, while the remaining six populations exhibited π values of 0 (Table 1).

**TABLE 1:** Haplotype frequencies and nucleotide diversities (π) of Chinese Asian elephant populations.

Haplotype	Yexianggu (*N* = 60)	Kongge (*N* = 9)	Mangla (*N* = 9)	Manga (*N* = 9)	Mangman (*N* = 12)	Puwen (*N* = 3)	Nangunhe (*N* = 19)
Haplotype 1	93.3%	100%	100%	100%	100%	100%	0
Haplotype 2	0	0	0	0	0	0	100%
Haplotype 3	3.3%	0	0	0	0	0	0
Haplotype 4	1.7%	0	0	0	0	0	0
Haplotype 5	1.7%	0	0	0	0	0	0
π	1.74050 ± 1.13893	0	0	0	0	0	0

### 3.3 Identification of Kinship Between Asian Elephant Individuals

The results revealed that parent‐offspring relationships were identified in the populations of Kongge (three pairs), Menga (one pair), Mengla (one pair), Mengman (three pairs), Nangunhe (10 pairs), and Yexianggu (102 pairs). Additionally, full‐sibling relationships were detected in the population of Mengla (three pairs), Mengman (six pairs), Nangunhe (four pairs), Puwen (one pair), and Yexianggu (31 pairs), and half‐sibling relationships were detected exclusively in Mengla (one pair) and Yexianggu (43 pairs).

### 3.4 Genetic Diversity of Asian Elephant Population

A total of 123 alleles were detected across all studied populations. The results demonstrated that the average Ho was lower than the average He in all Asian elephant populations (Table 2). The polymorphism information content (PIC) was highest in the Nangunhe population, followed by the Menga and Yexianggu populations, whereas the Mengla population showed the lowest PIC value (Table 2).

**TABLE 2:** Statistical analysis of 10 microsatellite loci of the six Asian elephant populations.

Locus	Yexianggu (*N* = 60)			Mengman (*N* = 12)			Kongge (*N* = 9)			Mengla (*N* = 9)			Menga (*N* = 9)			Nangunhe (*N* = 19)		
	Ho	He	PIC	Ho	He	PIC	Ho	He	PIC	Ho	He	PIC	Ho	He	PIC	Ho	He	PIC
LafMS09	0.483	0.613	0.558	0.750	0.761	0.691	0.333	0.582	0.448	0.000	0.523	0.438	0.500	0.833	0.755	0.474	0.740	0.694
LA2	0.439	0.597	0.568	0.455	0.723	0.627	0.286	0.703	0.599	0.444	0.634	0.539	0.333	0.837	0.759	0.789	0.795	0.741
EMU01	0.305	0.478	0.423	0.417	0.685	0.589	0.889	0.810	0.728	0.125	0.692	0.592	0.111	0.542	0.426	0.158	0.485	0.449
EMU04	0.433	0.561	0.507	0.250	0.518	0.408	0.444	0.366	0.286	0.000	0.000	0.000	0.600	0.711	0.581	0.563	0.772	0.707
EMU07	0.797	0.770	0.730	0.909	0.814	0.742	0.571	0.725	0.632	0.889	0.634	0.539	0.286	0.571	0.483	0.368	0.700	0.644
EMU11	0.700	0.603	0.560	0.583	0.482	0.425	0.556	0.621	0.501	0.111	0.111	0.099	0.333	0.386	0.327	0.632	0.590	0.511
EMU12	0.633	0.714	0.671	0.200	0.600	0.492	0.222	0.471	0.409	0.333	0.307	0.269	0.571	0.725	0.617	0.556	0.732	0.657
EMU15	0.633	0.604	0.542	0.500	0.540	0.420	0.333	0.425	0.321	0.400	0.733	0.610	0.556	0.830	0.749	0.263	0.330	0.304
EMU17	0.532	0.724	0.671	0.250	0.540	0.444	0.571	0.484	0.406	0.400	0.711	0.563	0.333	0.636	0.530	0.333	0.560	0.445
FH60	0.491	0.651	0.582	0.500	0.592	0.510	0.333	0.333	0.239	0.222	0.216	0.194	0.222	0.712	0.613	0.737	0.798	0.742
Average	0.545	0.632	0.581	0.481	0.626	0.535	0.454	0.552	0.457	0.292	0.456	0.384	0.385	0.678	0.584	0.487	0.650	0.589

Abbreviations: He, expected heterozygosity; Ho, observed heterozygosity; PIC, polymorphic information content.

The results of the genetic diversity indices indicated that the average number of alleles was 8.6 in the Yexianggu population, followed by the Nangunhe (5.6) and Menga (4.5) populations, whereas the Puwen population displayed the lowest number of alleles (2.5). The average number of private alleles exhibited considerable variation across the seven Asian elephant populations, ranging from 0 (Kongge) to 23 (Yexianggu). The He for these populations ranged from 0.46 (Mengla) to 0.68 (Menga), while the Ho ranged from 0.29 (Mengla) to 0.54 (Yexianggu). The *F*_IS_ of the Yexianggu (0.06) population was the lowest, whereas that of the Menga population (0.35) was the highest. The average number of microsatellite variance ranged from 5.89 (Mengla) to 12.84 (Nangunhe) (Table 3).

**TABLE 3:** Statistics of genetic diversity parameters for seven Asian elephant populations in China.

	Yexianggu (*N* = 60)	Mengman (*N* = 12)	Kongge (*N* = 9)	Mengla (*N* = 9)	Menga (*N* = 9)	Nangunhe (*N* = 60)
The average number of alleles	8.6	4.1	3.3	3.1	4.5	5.6
Number of private alleles	23	3	0	8	7	9
The average number of *F*_IS_	0.06	0.09	0.18	0.30	0.35	0.31
The average number of microsatellite variance	7.34	8.10	6.83	5.89	11.86	12.84

Abbreviations: *F*_IS_, inbreeding coefficient; He, expected heterozygosity; Ho, observed heterozygosity.

### 3.5 Hardy–Weinberg Equilibrium of the Asian Elephant Populations in China

A Chi‐square test was conducted to evaluate whether the genotype frequencies at 10 microsatellite loci conformed to Hardy–Weinberg equilibrium (HWE) in each population. The results indicated that the Yexianggu population significantly deviated from HWE at all 10 microsatellite loci (*p* < 0.05). The Puwen and Kongge populations exhibited significant deviations from HWE at one microsatellite locus, namely E17 and E11, respectively (*p* < 0.05). The Mengman population significantly deviated from HWE at four loci (L2, E1, E17, and F60; *p* < 0.05). Similarly, deviations were observed at three loci (L9, E1, and E15) in the Mengla population, and at five loci (L2, E1, E15, E17, and F60; *p* < 0.05) in the Menga population. The Nangunhe population also showed significant deviations at loci L9, E1, E4, and E7 (*p* < 0.05) (Table S3).

### 3.6 Individual Inbreeding and Pairwise Relatedness in Asian Elephant Populations

**FIGURE 4:**
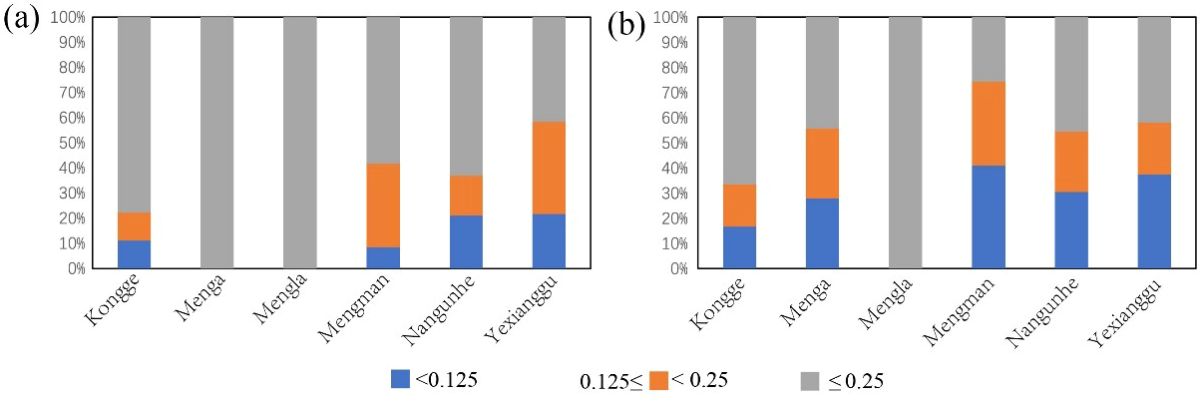
The distributions of (a) estimated individual inbreeding coefficients and (b) estimated pairwise relatedness values.

The inbreeding coefficient and pairwise relatedness were calculated based on the studied microsatellite loci. The Puwen population was excluded from this analysis due to insufficient sample size for robust genetic analysis (*N* = 3). The results showed that the average inbreeding coefficients for the Kongge, Menga, Mengla, Mengman, Nangunhe, and Yexianggu populations were 0.422, 0.521, 0.668, 0.350, 0.351, and 0.244, respectively. More than 50% of individuals in five populations (Kongge, Menga, Mengla, Mengman, and Nangunhe) exhibited inbreeding coefficients exceeding 0.25, while 42% of individuals in the Yexianggu population also surpassed this threshold (Figure 4a). The proportion of individuals with *f*_*M*_ below 0.125 was comparatively low, accounting for 11%, 0%, 0%, 8%, 21%, and 22% of the Kongge, Menga, Mengla, Mengman, Nangunhe, and Yexianggu populations, respectively (Figure 4a). Genetic analysis showed that the average pairwise relatedness was 0.346, 0.256, 0.577, 0.228, 0.256, and 0.234 for the Kongge, Menga, Mengla, Mengman, Nangunhe, and Yexianggu populations, respectively. Among them, 17%, 28%, 0%, 41%, 30%, and 37% of individuals in Kongge, Menga, Mengla, Mengman, Nangunhe, and Yexianggu populations exhibited *R* values less than 0.125, respectively (Figure 4b).

Genetic distance calculations among the seven populations revealed that the Mengla population exhibited the greatest genetic divergence from all others, whereas the smallest genetic distance was observed between the Mengman and Kongge populations (Table 4).

**TABLE 4:** The pairwise distance of different Asian elephant populations.

	Yexianggu	Mengman	Puwen	Kongge	Mengla	Menga	Nangunhe
Yexianggu		0.00	0.02	0.00	0.00	0.00	0.00
Mengman	0.08		0.06	0.05	0.00	0.00	0.00
Puwen	0.15	0.12		0.01	0.00	0.01	0.00
Kongge	0.18	0.06	0.24		0.00	0.00	0.00
Mengla	0.35	0.33	0.45	0.25		0.00	0.00
Menga	0.24	0.25	0.31	0.21	0.26		0.00
Nangunhe	0.12	0.21	0.20	0.23	0.38	0.25	

*Note:* Lower‐triangular and Upper‐triangular data matrix is the *F*_ST_ and *p* value, respectively.

Abbreviation: *F*_ST_, fixation index.

AMOVA results indicated that the highest relatedness (*R*_st_) was observed in the second group based on microsatellite data, but the highest *R*_st_ was observed in the first group based on mitochondrial DNA data (Table 5).

**TABLE 5:** Geographic subdivision analysis based on mtDNA and microsatellite data.

Subdivision	Sub‐groups	Microsatellite *R*_st_	MtDNA *F*_ST_
Two populations	[Nangunhe] [Yexianggu, Mengman, Puwen, Kongge, Mengla, Menga]	0.07	0.94
Two populations	[Mengla] [Nangunhe, Yexianggu, Mengman, Puwen, Kongge, Menga]	0.26	0.04
Three populations	[Nangunhe] [Mengla] [Yexianggu, Mengman, Puwen, Kongge, Menga]	0.22	0.93
Seven populations	[Nangunhe], [Yexianggu], [Mengman], [Puwen], [Kongge], [Mengla], [Menga]	0.24	0.86

*Note:* Subspecies was grouped by brackets into populations.

Abbreviations: *F*_ST_, fixation index; *R*_st_, relatedness.

### 3.7 The Bottleneck Effect in Asian Elephant Populations

Three microsatellite mutation models were used to analyze the bottleneck effect across six Asian elephant populations at 10 microsatellite loci, namely the Infinite Alleles Model (IAM), Stepwise Mutation Model (SMM), and Two‐Phase Model (TPM). The Puwen population was excluded due to an insufficient sample size. It was noted that these three models did not yield consistent results, which is a common occurrence due to their differing underlying assumptions. The results showed that Ho was lower than He at seven microsatellite loci (L9, L2, E1, E4, E12, E17, and F60) in the Yexianggu and Mengman populations, but the *P*‐values differed across the three models (Tables S4 and S5). The similar patterns were observed in other populations. In the Kongge population, the Ho was less than He at loci L9, L2, E7, E12, and F60 (Table S6). The Mengla population showed this pattern at eight microsatellite loci, with the exception of E4 and E7 (Table S7). Additionally, the Ho was less than He at all microsatellite loci in the Menga population (Table S8), and at L9, E1, E4, E7, E12, E15, and E17 in the Nangunhe population (Table S9). We prioritized the findings under the TPM and SMM models, as they are biologically more realistic for microsatellite loci than the IAM model (Peery et al. 2012). In summary, both the Sign test and Wilcoxon test under the SMM and TPM models indicated a significant heterozygosity excess in the Yexianggu population (Table S10).

### 3.8 Phylogenetic Relationships of Asian Elephant Populations

To investigate the phylogenetic relationships among the seven Asian elephant populations, a Neighbor‐Joining (NJ) tree was constructed based on genetic distances derived from the proportion of shared alleles (*D*_ps_) and the kinship coefficient (*D*_kf_). The phylogenetic tree indicated that most individuals from Yexianggu clustered with those from Puwen (2 individuals), Mengman (7 individuals), and Menga (1 individual). All Mengla individuals formed a single cluster. The individuals from Nangunhe were divided into two clades, with one clade containing an individual from Yexianggu, and the other containing individuals from Kongge, Mengman, Puwen, Menga, and Yexianggu populations (Figure 3).

### 3.9 Population Structure of Asian Elephants

The population structure of Asian elephants was analyzed to examine their genetic relationships. The results of STRUCTURE showed that the log‐likelihood value of the data (Ln Probability) was highest when *K* = 11 (Figure S1). The analysis revealed that the Mengla population shared a common ancestor at *K* = 11. In the Yexianggu population, the majority of individuals shared a common ancestor (Str_8), which was admixed with three potential ancestors (Str_4, Str_9, and Str_10), indicating high genetic variability. The individuals from the Nangunhe population were divided into two genetic clusters. Furthermore, the six individuals from the Menga population shared a common ancestor (Str_7). Two individuals from Puwen and four individuals from Mengman may have shared a common ancestor (Str_3), while the other three individuals from Mengman shared a similar ancestor (Str_11). These results were consistent with the phylogenetic relationships among individuals from the different Asian elephant populations (Figure 3).

## 4 Discussion

In the Asian elephant populations studied, the microsatellite loci showed moderate polymorphism in four populations (Nangunhe, Menga, Yexianggu, and Mengman), with their PIC marginally exceeding the threshold for high polymorphism (PIC > 0.5). This indicates that these populations exhibited higher genetic polymorphism compared to other Asian elephant populations. Among them, the Yexianggu population exhibited the highest genetic diversity, which may provide greater adaptive potential to diverse environmental conditions and pressures (Tables 1, 2, 3). However, future assessments of fitness and adaptability, such as long‐term population growth rates and individual survival data, are needed to robustly evaluate this adaptive advantage. In contrast, the Kongge population displayed a limited number of alleles and an absence of private alleles, suggesting constrained genetic diversity. However, these results may have been influenced by the relatively small sample size (*N* = 9) and potential genotyping inaccuracies.

Although the Yexianggu population maintains relatively high genetic diversity, a significant excess of heterozygotes has been observed, likely resulting from recent gene flow with a previously isolated population. Additionally, this population has historically undergone a genetic bottleneck followed by a decline in population size. This indicates that, compared with other Asian elephant populations in history, the adaptive capacity of the Yexianggu population to environmental changes may still require enhancement. The Ho was lower than the He in this population further supporting this conclusion from another perspective. Notably, the heterozygote deficiency (Ho<He) observed in the Yexianggu population can also be attributed to inbreeding.

The He was greater than the Ho in the studied Chinese Asian elephant populations. This phenomenon may be attributed to two main factors. Firstly, the populations of Yexianggu, Mengman, Mengla, Menga, and Nangunhe exhibit varying degrees of inbreeding. Among them, the *F*_IS_ (0.06) of the Yexianggu population was the lowest, suggesting a low inbreeding coefficient and relatively high genetic diversity. In contrast, the *F*_IS_ (0.31) of the Nangunhe population was relatively high, second only to that of the Menga population (0.30), suggesting severe inbreeding (Table 3). These findings were further supported by the results of individual inbreeding coefficients and estimates of pairwise relatedness. Therefore, the presence of inbreeding within these Asian elephant populations may contribute to a lower Ho. Secondly, the geographical isolation of the Nangunhe population may restrict random mating among individuals, thereby exacerbating random fluctuations in gene frequencies. Other Asian elephant populations may also be affected by genetic drift, a reduction in observed heterozygosity.

Consistent with other studies on the genetic diversity of Asian elephants in China, we found that the Chinese Asian elephant population is divided into α and β clades. Among these, all individuals from the Nangunhe population exhibited haplotype 2, which belongs to the β clade. This may be attributed to geographical isolation between the Nangunhe and other Asian elephant populations, which hinders gene exchange. Furthermore, the fact that the other six Asian elephant populations shared haplotype 1 suggests that they may share a common ancestor or have experienced extensive gene exchange (Shi et al. 2025). The individuals from the Nangunhe population formed two distinct clusters. One contained six individuals, while the remaining individuals clustered with those from Yexianggu. This pattern suggests a relatively close genetic relationship between some individuals of the Nangunhe and Yexianggu populations. Notably, we observed that an individual (HM28) from the Yexianggu population clustered with 13 individuals from the Nangunhe population. To determine the origin of individual HM28, we verified with staff from the Xishuangbanna National Nature Reserve in Yunnan. It was eventually determined that HM28 originated in northern Myanmar, a region that borders the habitat of the Nangunhe population (Lincang City, Yunnan Province, China). This suggests that microsatellite and mitochondrial markers can be used as critical tools for species tracing, which demonstrates the accuracy and reliability of our study.

Geographically, the four populations (Yexianggu, Mengman, Kongge, and Puwen) are relatively close to each other compared with other populations. The results of *F*_ST_ also revealed lower genetic differentiation between these populations. Among them, the genetic distance between Mengla and other populations is the largest. Specifically, *F*_ST_ between Mengla and Nangunhe was 0.38, followed by 0.35 (Mengla–Yexianggu), and 0.33 (Mengla–Mengman). It can be seen that the genetic exchange between the Mengla and Nangunhe, Mengla and Mengman, Mengla and Yexianggu populations may have been limited for several reasons such as geographic isolation, habitat barriers, and anthropogenic factors. The NJ phylogenetic tree indicates that the 9 individuals of the Mengla population are clustered into a single branch. This evidence indicates that the Mengla population may belong to a genetically distinct population. To further verify this, we conducted AMOVA analysis to obtain accurate clustering results among different Asian elephant populations using microsatellite and mitochondrial data, and found that the results obtained from mitochondrial DNA and microsatellite markers were not completely consistent. Analysis of mitochondrial DNA supported the division of the seven Asian elephant populations into two management units, with the Nangunhe population considered an independent management unit. This primarily provides insights into maternal genetic inheritance (Hajibabaei et al. 2007). On the other hand, the results of microsatellites seem to indicate that the Mengla population should be managed as an independent management unit. We reasonably believe that these seven Asian elephant populations should be divided into three management units, namely unit 1 (Nangunhe), unit 2 (Mengla), and unit 3 (Yexianggu, Mengman, Puwen, Kongge, and Mengla). The biparental inheritance pattern of the nuclear genome permits a more comprehensive assessment of the population structure (Hajibabaei et al. 2007). This was also demonstrated by the phylogenetic trees among different individuals from seven Asian elephant populations. Additionally, as shown in Figure 1, the geographical distances among the five populations in unit 3 were closer, while the Nangunhe and Mengla populations were further away from unit 3, demonstrating the accuracy of the division of management units from the perspective of geographic context. The nuclear‐cytoplasmic inconsistency observed in this study provides potential evidence for gene flow among different Asian elephant populations in China.

Finally, we compared the genetic diversity of Asian elephants in this study with that of other wildlife in China. The genetic diversity of Asian elephants was lower than that observed in other wild mammals in China, including the giant panda (He = 0.685) (Dai et al. 2020; Zhang et al. 2007), pangolin (He = 0.895) (Gao et al. 2010), North China leopards (He = 0.734) (Yin et al. 2023), and Tibetan antelope (*Pantholops hodgsoni*, He = 0.821, π = 0.027) (Du et al. 2010). Compared with previous studies on the genetic diversity of Asian elephants, the nucleotide diversity of the Chinese Asian elephant population was found to be lower than that of populations in India (π = 0.012), Laos (π = 0.011), Vietnam (π = 0.006), and Sri Lanka (π = 0.016) (Prithiviraj et al. 2000; Sukumar 2006; Vidya et al. 2004, 2007; Ahlering, Hailer, et al. 2011).

In summary, our study reveals low genetic diversity and a certain degree of inbreeding in Chinese Asian elephant populations. To enhance regional genetic diversity, the primary conservation strategy should focus on establishing population connectivity within China to facilitate natural gene flow. For unit 3 (Yexianggu, Mengman, Puwen, Kongge, and Menga), which exhibits high habitat connectivity potential, national parks or ecological corridors (Gatti 2025; Jeong et al. 2024) should be established to promote the migration of Asian elephant individuals among these five populations, thereby enabling gene flow and enhancing the genetic diversity of Asian elephant populations in China. This is akin to the successful ecological corridors built for giant panda (Yin et al. 2006). For the domestic Asian elephant population, separate management measures should be formulated for the three management units to maintain existing genetic diversity. Among them, the Nangunhe population possesses a single haplotype. Introducing Asian elephant individuals from Yexianggu could be a viable method to enhance the genetic diversity of the Nangunhe population. This can also reduce the inbreeding degree within the Nangunhe population. However, reintroducing individuals is complex work, and such intervention must be carefully considered. This strategy is contingent upon and must be preceded by comprehensive assessments of habitat suitability in the Nangunhe and thorough evaluation of potential genetic risks to ensure its ecological and genetic feasibility. In addition, we cautiously recommend exploring the feasibility of introducing Asian elephant individuals with higher genetic diversity from neighboring countries, including India, Laos, Vietnam, and Sri Lanka. However, any such strategy must be preceded by a rigorous assessment of ecological compatibility, potential disease risks, and the establishment of necessary international policy frameworks to ensure its safety and success. Continuous genetic monitoring of the Chinese Asian elephant population is also one of the measures of scientific conservation, which can help researchers understand the level of genetic diversity and adjust conservation and management measures accordingly. Therefore, we recommend implementing a long‐term genetic monitoring program at regular intervals (every year) using non‐invasive samples (feces) and standardized molecular methods (mitochondrial DNA and microsatellites). Furthermore, the government should further strengthen the enforcement of policies related to the protection of Asian elephants to raise conservation awareness among local communities.

Our study explored the genetic diversity and population structure of the Chinese Asian elephant population, providing a solid scientific basis for identifying and delineating priority conservation units, designing effective ecological corridors, facilitating individual migrations, and promoting gene flow among populations in China. However, protecting the flagship species of the Asian elephant is a challenging and long‐term endeavor. There is still a long way to go in this regard.

## 5 Conclusion

In summary, we analyzed the genetic diversity of seven Chinese Asian elephant populations using microsatellite and mitochondrial DNA. Our study showed that the genetic diversity of these populations remains low, although the average PIC at 10 microsatellite loci exceeded 0.5 in the Yexianggu, Nangunhe, and Menga populations. Among these, the Yexianggu population has experienced a bottleneck and shows significant heterozygosity excess. Furthermore, the Menga population exhibited the highest level of genetic divergence from the other populations. Based on the two genetic markers, we divided the seven Asian elephant populations into three management units, including unit 1 (Nangunhe), unit 2 (Mengla), and unit 3 (Yexianggu, Mengman, Puwen, Kongge, and Menga). This study will contribute to the sustainable management of Chinese Asian elephant populations in the future.

## Author Contributions

**Xing Yun:** investigation (equal). **Jingshan Wang:** investigation (equal). **Xu Li:** project administration (equal). **Bin Wang:** resources (equal). **Shaobing Yang:** investigation (supporting). **Dusu Wen:** writing – review and editing (supporting). **Weibin Wang:** resources (equal). **Ruobing Han:** conceptualization (equal), data curation (equal), formal analysis (equal), investigation (equal), writing – original draft (equal), writing – review and editing (equal).

## Ethics Statement

All experimental designs and animal handling were approved by the Institutional Animal Care and Use Committee of Southwest Forestry University, China.

## Conflicts of Interest

The authors declare no conflicts of interest.

## Supporting information


**Figure S1:** STRUCTURE output summary charts.

**Table S1:** The microsatellite loci in this study.
**TABLE S2:** Statistics of Null Allele and ADO in the Chinese Asian elephant Population.
**TABLE S3:** Hardy–Weinberg equilibrium test for seven Asian elephant population in China.
**TABLE S3:** Hardy–Weinberg equilibrium test for seven Asian elephant population in China.
**TABLE S4:** The bottleneck test by microsatellite in the population of Yexianggu.
**TABLE S5:** The bottleneck test by microsatellite in the population of Mengman.
**TABLE S6:** The bottleneck test by microsatellite in the population of Kongge.
**TABLE S7:** The bottleneck test by microsatellite in the population of Mengla.
**TABLE S8:** The bottleneck test by microsatellite in the population of Menga.
**TABLE S9:** The bottleneck test by microsatellite in the population of Nangunhe.
**TABLE S10:** Sign test and Wilcoxon signed‐rank test to evaluate Jining Qing goat for mutation drift equilibrium under different models.

## Data Availability

All the required data is uploaded as Supporting Information.
