# Supplementary figures and images for: Unraveling the Genetic Diversity of Asian Elephants (Elephas maximus) in China: Implications for the Conservation of Asian Elephants
Source: Ecol Evol. 2025 Nov 14;15(11):e72498. doi: 10.1002/ece3.72498 (PMC12617253; doi:10.1002/ece3.72498)

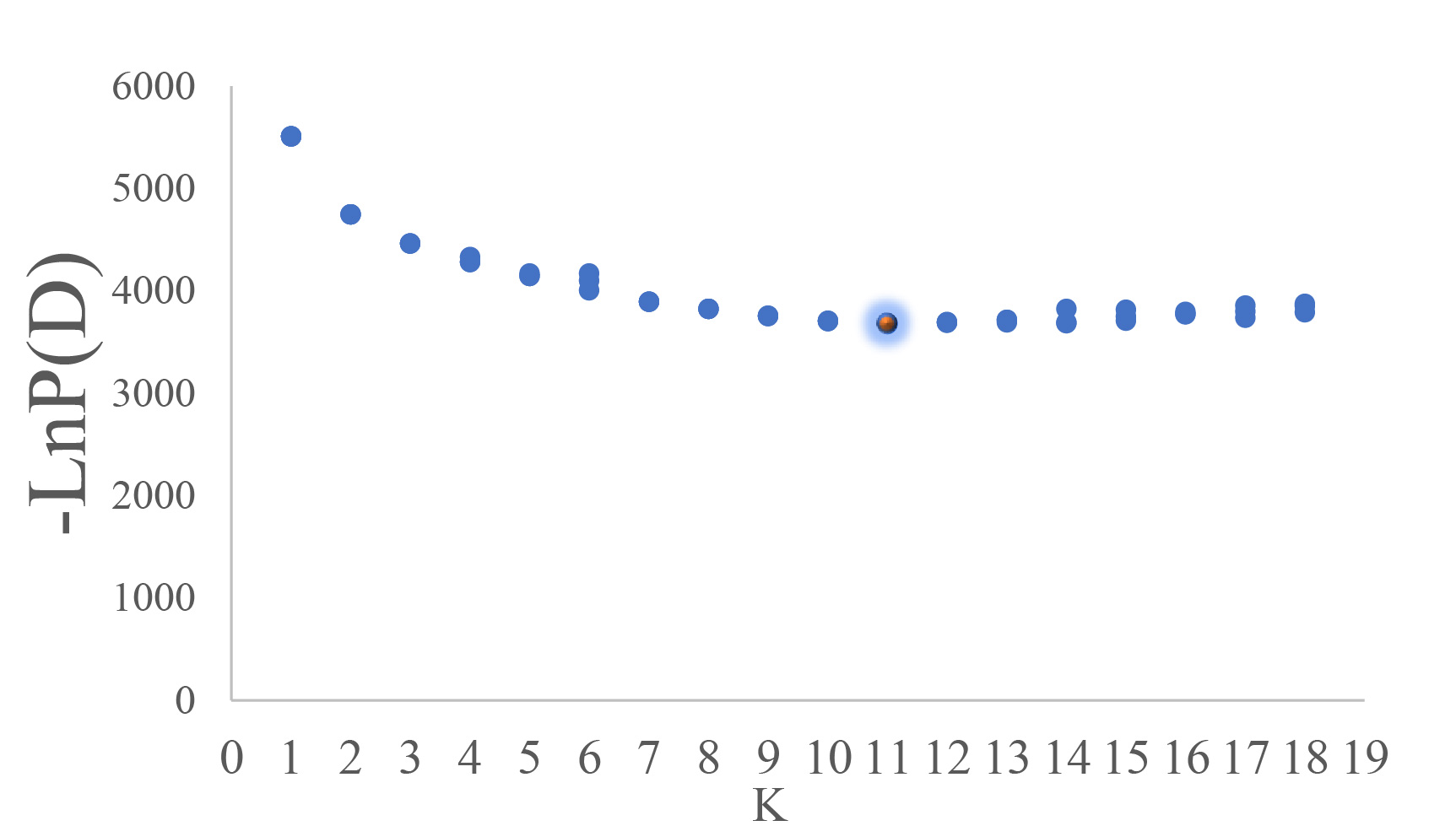

Supplement: Supplementary file 1 — Figure S1: STRUCTURE output summary charts. [file ECE3-15-e72498-s002.jpg]
