# Supplementary material for: Unraveling the Genetic Diversity of Asian Elephants (Elephas maximus) in China: Implications for the Conservation of Asian Elephants
Source: Ecol Evol. 2025 Nov 14;15(11):e72498. doi: 10.1002/ece3.72498 (PMC12617253; doi:10.1002/ece3.72498)
Supplement: Supplementary file 2 — Table S1: The microsatellite loci in this study. TABLE S2: Statistics of Null Allele and ADO in the Chinese Asian elephant Population. TABLE S3: Hardy–Weinberg equilibrium test for seven Asian elephant population in China. TABLE S3: Hardy–Weinberg equilibrium test for seven Asian elephant population in China. TABLE S4: The bottleneck test by microsatellite in the population of Yexianggu. TABLE S5: The bottleneck test by microsatellite in the population of Mengman. TABLE S6: The bottleneck test by microsatellite in the population of Kongge. TABLE S7: The bottleneck test by microsatellite in the population of Mengla. TABLE S8: The bottleneck test by microsatellite in the population of Menga. TABLE S9: The bottleneck test by microsatellite in the population of Nangunhe. TABLE S10: Sign test and Wilcoxon signed‐rank test to evaluate Jining Qing goat for mutation drift equilibrium under different models. [file ECE3-15-e72498-s001.docx]

Table S1 The microsatellite loci in this study.

| Locus | Repeat motif | Primer sequence (5’-3’) | *T*_a_℃ |
| --- | --- | --- | --- |
| EMU01 | (GTT)_14_ | F：AGGACTTATTTGCTTAGATGG  R：AGGCAATGTTTCGTTCTGT | 58 |
| EMU04 | (TG)_12_ | F：TGACTCTCCCTCTTCTGCAT  R：GGCTGAGAGGGAAAGAAATTG | 58 |
| EMU07 | (TG)_15_ | F：GAGCAGTGCCTTTCGTGAC  R：AGCCTGGGAGGTAAGTAGCA | 58 |
| EMU11 | (TG)_9_ | F：CAATATGGGTGTGGGTTTCC  R: GAAATGCAGCATAAATAATATCATGG | 58 |
| EMU12 | (AC)_8_ | F：CCAAAGAAGACCCATGTTCC  R：CTGACTATGGGGGAGACTGC | 58 |
| EMU15 | (AC)14 | F：TTCGGGATGTTCTCTTCTGT  R：GGGGCTTAACTAATAGGCTTCA | 58 |
| EMU17 | (GT)16 | F：CACTCAGAGTTCCAAGAAGCAG  R：TGCCAGCCATTTCCTCTC | 58 |
| LA2 | (CA)6(CGTA)2 (CA)6 | F：CTTGGTGGGAGTCATGACCT  R：GGAGAAATGACTGCCCGATA | 58 |
| LafMS09 | (GATA)2 (GACA)7 | F：CTGGGGCAGTAAGCTGTATTTATC  R：ACGAGGATGACAGACCAGGCAACA | 58 |
| FH60 | (CA)13 | F：CAAGAAGCTTTGGGATTGGG  R：CCTGCAGCTCAGAACACCTG | 61 |

Table S2 Statistics of Null Allele and ADO in the Chinese Asian elephant Population

|  | Yexianggu (N=60) | | Mengman (N=12) | | Kongge (N=9) | | Mengla (N=9) | | Menga (N=9) | | Nangunhe (N=19) | |
| --- | --- | --- | --- | --- | --- | --- | --- | --- | --- | --- | --- | --- |
|  | Null Allele | ADO | Null Allele | ADO | Null Allele | ADO | Null Allele | ADO | Null Allele | ADO | Null Allele | ADO |
| LafMS09 | 0.114 | 0 | -0.0141 | 0 | 0 | 0 | 1 | 0 | 0.220 | 0 | 0.207 | 0 |
| LA2 | 0.149 | 0 | 0.2058 | 0 | 0 | 0 | 0.148 | 0 | 0.407 | 0 | -0.010 | 0 |
| EMU01 | 0.217 | 0 | 0.2233 | 0 | 0 | 0 | 0.677 | 0 | 0.644 | 0 | 0.499 | 0 |
| EMU04 | 0.125 | 0 | 0.3302 | 0 | 0 | 0 | 0 | 0 | 0.032 | 0 | 0.142 | 0 |
| EMU07 | -0.021 | 0 | -0.078 | 0 | 0 | 0 | -0.195 | 0 | 0.300 | 0 | 0.298 | 0 |
| EMU11 | -0.079 | 0 | -0.116 | 0 | 0 | 0 | -0.029 | 0 | 0.044 | 0 | -0.047 | 0 |
| EMU12 | 0.056 | 0 | 0.481 | 0 | 0 | 0 | -0.069 | 0 | 0.082 | 0 | 0.123 | 0 |
| EMU15 | -0.023 | 0 | 0.017 | 0 | 0 | 0 | 0.245 | 0 | 0.171 | 0 | 0.100 | 0 |
| EMU17 | 0.148 | 0 | 0.348 | 0 | 0 | 0 | 0.231 | 0 | 0.273 | 0 | 0.241 | 0 |
| FH60 | 0.136 | 0 | 0.052 | 0 | 0 | 0 | -0.044 | 0 | 0.503 | 0 | 0.027 | 0 |
| Average | 0.082 | 0 | 0.145 | 0 | 0 | 0 | 0.196 | 0 | 0.268 | 0 | 0.158 | 0 |

ADO: allele dropout

Table S3 Hardy-Weinberg equilibrium test for seven Asian elephant population in China

|  | L9 | L2 | E1 | E4 | E7 | E11 | E12 | E15 | E17 | F60 |
| --- | --- | --- | --- | --- | --- | --- | --- | --- | --- | --- |
| Yexianggu | 0.0000 | 0.000000 | 0.0000 | 0.0000 | 0.0000 | 0.05004 | 0.0000 | 0.0030 | 0.0000 | 0.0000 |
| Mengman | 0.8249 | 0.0236 | 0.0088 | 0.1340 | 0.0781 | 0.9452 | 0.0818 | 0.8481 | 0.0000 | 0.0138 |
| Puwen | 0.3208 | 1 | 0.5637 | 0.3208 | 0.5724 | 0.5637 | 1 | 0.5637 | 0.0455 | 1 |
| Kongge | 0.3146 | 0.0579 | 0.6634 | 0.4677 | 0.7609 | 0.0006 | 0.0744 | 0.4784 | 0.8496 | 1 |
| Mengla | 0.0000 | 0.3554 | 0.0022 | - | 0.5467 | 1.0000 | 0.9729 | 0.0430 | 0.0719 | 0.9955 |
| Menga | 0.0552 | 0.0006 | 0.0149 | 0.5184 | 0.1799 | 0.1710 | 0.8153 | 0.0055 | 0.0307 | 0.0088 |
| Nangunhe | 0.0003 | 0.5343 | 0.0000 | 0.0000 | 0.0000 | 0.5327 | 0.4345 | 0.0630 | 0.2200 | 0.1089 |

Table S4 The bottleneck test by microsatellite in the population of Yexianggu

| Locus | Ho | *IAM* | | *SMM* | | *TPM* | |
| --- | --- | --- | --- | --- | --- | --- | --- |
|  |  | *Heq* | *Prob* | *Heq* | *Prob* | *Heq* | *Prob* |
| L9 | 0.483 | 0.766 | 0.0710 | 0.870 | 0.0000 | 0.829 | 0.0030 |
| L2 | 0.439 | 0.716 | 0.1290 | 0.841 | 0.0000 | 0.793 | 0.0110 |
| E1 | 0.305 | 0.608 | 0.1860 | 0.766 | 0.0020 | 0.696 | 0.0400 |
| E4 | 0.433 | 0.558 | 0.4260 | 0.721 | 0.0330 | 0.651 | 0.1700 |
| E7 | 0.797 | 0.691 | 0.2420 | 0.821 | 0.0980 | 0.766 | 0.4460 |
| E11 | 0.700 | 0.503 | 0.3420 | 0.671 | 0.1900 | 0.588 | 0.4730 |
| E12 | 0.633 | 0.745 | 0.2930 | 0.855 | 0.0000 | 0.808 | 0.0700 |
| E15 | 0.633 | 0.552 | 0.4560 | 0.721 | 0.0650 | 0.642 | 0.2800 |
| E17 | 0.532 | 0.673 | 0.4330 | 0.799 | 0.0690 | 0.741 | 0.3340 |
| F60 | 0.491 | 0.772 | 0.0790 | 0.871 | 0.0000 | 0.830 | 0.0080 |

IAM: infinite alleles model, SMM: stepwise mutation model, TPM: two-phase model, Ho: The observed heterozygosity, Heq: The expected heterozygosity, Prob: The probability.

Table S5 The bottleneck test by microsatellite in the population of Mengman

| Locus | Ho | IAM | | SMM | | TPM | |
| --- | --- | --- | --- | --- | --- | --- | --- |
|  |  | *Heq* | *Prob* | *Heq* | *Prob* | *Heq* | *Prob* |
| L9 | 0.750 | 0.765 | 0.4180 | 0.824 | 0.0720 | 0.796 | 0.2160 |
| L2 | 0.455 | 0.570 | 0.0830 | 0.650 | 0.2450 | 0.610 | 0.1460 |
| E1 | 0.417 | 0.560 | 0.1960 | 0.650 | 0.4470 | 0.607 | 0.3150 |
| E4 | 0.250 | 0.433 | 0.4120 | 0.521 | 0.4170 | 0.475 | 0.5030 |
| E7 | 0.909 | 0.659 | 0.0140 | 0.733 | 0.0520 | 0.703 | 0.0410 |
| E11 | 0.583 | 0.533 | 0.2940 | 0.648 | 0.0670 | 0.605 | 0.1620 |
| E12 | 0.200 | 0.578 | 0.4890 | 0.663 | 0.2060 | 0.622 | 0.3370 |
| E15 | 0.500 | 0.442 | 0.3710 | 0.529 | 0.4480 | 0.481 | 0.4500 |
| E17 | 0.250 | 0.439 | 0.3520 | 0.523 | 0.4810 | 0.478 | 0.4500 |
| F60 | 0.500 | 0.616 | 0.3950 | 0.677 | 0.1620 | 0.644 | 0.2760 |

IAM: infinite alleles model, SMM: stepwise mutation model, TPM: two-phase model, Ho: The observed heterozygosity, Heq: The expected heterozygosity, Prob: The probability.

Table S6 The bottleneck test by microsatellite in the population of Kongge

| Locus | Ho | IAM | | SMM | | TPM | |
| --- | --- | --- | --- | --- | --- | --- | --- |
|  |  | *Heq* | *Prob* | *Heq* | *Prob* | *Heq* | *Prob* |
| L9 | 0.333 | 0.463 | 0.2970 | 0.547 | 0.4950 | 0.505 | 0.3660 |
| L2 | 0.286 | 0.639 | 0.3570 | 0.694 | 0.4350 | 0.663 | 0.4280 |
| E1 | 0.889 | 0.692 | 0.0520 | 0.746 | 0.1320 | 0.722 | 0.0750 |
| E4 | 0.444 | 0.283 | 0.3760 | 0.322 | 0.5050 | 0.314 | 0.4800 |
| E7 | 0.571 | 0.733 | 0.4500 | 0.766 | 0.2090 | 0.750 | 0.3500 |
| E11 | 0.556 | 0.466 | 0.1750 | 0.545 | 0.3410 | 0.517 | 0.2640 |
| E12 | 0.222 | 0.588 | 0.2030 | 0.671 | 0.0340 | 0.637 | 0.0900 |
| E15 | 0.333 | 0.279 | 0.2890 | 0.316 | 0.3790 | 0.305 | 0.3570 |
| E17 | 0.571 | 0.497 | 0.4800 | 0.568 | 0.2630 | 0.533 | 0.3700 |
| F60 | 0.333 | 0.433 | 1.0000 | 0.458 | 0.4440 | 0.445 | 0.4990 |

IAM: infinite alleles model, SMM: stepwise mutation model, TPM: two-phase model, Ho: The observed heterozygosity, Heq: The expected heterozygosity, Prob: The probability.

Table S7 The bottleneck test by microsatellite in the population of Mengla

| Locus | Ho | IAM | | SMM | | TPM | |
| --- | --- | --- | --- | --- | --- | --- | --- |
|  |  | *Heq* | *Prob* | *Heq* | *Prob* | *Heq* | *Prob* |
| L9 | 0.000 | 0.470 | 0.4670 | 0.535 | 0.3990 | 0.504 | 0.4680 |
| L2 | 0.444 | 0.605 | 0.5020 | 0.668 | 0.3030 | 0.631 | 0.4380 |
| E1 | 0.125 | 0.615 | 0.3240 | 0.679 | 0.6010 | 0.652 | 0.4630 |
| E4 | 0.000 | - | - | - | - | - | - |
| E7 | 0.889 | 0.597 | 0.4810 | 0.667 | 0.3020 | 0.637 | 0.4240 |
| E11 | 0.111 | 0.290 | 0.2940 | 0.333 | 0.2090 | 0.314 | 0.2590 |
| E12 | 0.333 | 0.468 | 0.2470 | 0.541 | 0.0900 | 0.500 | 0.1620 |
| E15 | 0.400 | 0.701 | 0.4980 | 0.736 | 0.6740 | 0.720 | 0.5910 |
| E17 | 0.400 | 0.563 | 0.0730 | 0.603 | 0.1420 | 0.596 | 0.1090 |
| F60 | 0.222 | 0.467 | 0.1230 | 0.539 | 0.0310 | 0.503 | 0.0690 |

IAM: infinite alleles model, SMM: stepwise mutation model, TPM: two-phase model, Ho: The observed heterozygosity, Heq: The expected heterozygosity, Prob: The probability.

Table S8 The bottleneck test by microsatellite in the population of Menga

| Locus | Ho | IAM | | SMM | | TPM | |
| --- | --- | --- | --- | --- | --- | --- | --- |
|  |  | *Heq* | *Prob* | *Heq* | *Prob* | *Heq* | *Prob* |
| L9 | 0.500 | 0.823 | 0.5760 | 0.857 | 0.2250 | 0.843 | 0.3520 |
| L2 | 0.333 | 0.751 | 0.0880 | 0.805 | 0.3070 | 0.781 | 0.1700 |
| E1 | 0.111 | 0.465 | 0.4120 | 0.540 | 0.4410 | 0.513 | 0.5390 |
| E4 | 0.600 | 0.700 | 0.6480 | 0.735 | 0.3110 | 0.721 | 0.3950 |
| E7 | 0.286 | 0.637 | 0.2590 | 0.693 | 0.1040 | 0.664 | 0.1790 |
| E11 | 0.333 | 0.469 | 0.3240 | 0.550 | 0.1280 | 0.516 | 0.1990 |
| E12 | 0.571 | 0.725 | 0.4980 | 0.776 | 0.2060 | 0.754 | 0.3370 |
| E15 | 0.556 | 0.689 | 0.0090 | 0.745 | 0.0350 | 0.724 | 0.0250 |
| E17 | 0.333 | 0.664 | 0.3520 | 0.710 | 0.1690 | 0.685 | 0.2530 |
| F60 | 0.222 | 0.598 | 0.1640 | 0.671 | 0.3740 | 0.639 | 0.2620 |

IAM: infinite alleles model, SMM: stepwise mutation model, TPM: two-phase model, Ho: The observed heterozygosity, Heq: The expected heterozygosity, Prob: The probability.

Table S9 The bottleneck test by microsatellite in the population of Nangunhe

| Locus | Ho | IAM | | SMM | | TPM | |
| --- | --- | --- | --- | --- | --- | --- | --- |
|  |  | *Heq* | *Prob* | *Heq* | *Prob* | *Heq* | *Prob* |
| L9 | 0.474 | 0.788 | 0.1960 | 0.853 | 0.0050 | 0.823 | 0.0590 |
| L2 | 0.789 | 0.718 | 0.2230 | 0.798 | 0.3830 | 0.761 | 0.3770 |
| E1 | 0.158 | 0.598 | 0.2040 | 0.701 | 0.0220 | 0.651 | 0.0800 |
| E4 | 0.563 | 0.732 | 0.3990 | 0.806 | 0.1780 | 0.776 | 0.3810 |
| E7 | 0.368 | 0.664 | 0.4560 | 0.758 | 0.1420 | 0.714 | 0.3290 |
| E11 | 0.632 | 0.396 | 0.1160 | 0.487 | 0.2270 | 0.443 | 0.1810 |
| E12 | 0.556 | 0.604 | 0.1390 | 0.704 | 0.4330 | 0.660 | 0.2520 |
| E15 | 0.263 | 0.512 | 0.1620 | 0.623 | 0.0250 | 0.561 | 0.0830 |
| E17 | 0.333 | 0.392 | 0.1850 | 0.500 | 0.4040 | 0.443 | 0.2450 |
| F60 | 0.737 | 0.716 | 0.1790 | 0.795 | 0.4370 | 0.761 | 0.3370 |

IAM: infinite alleles model, SMM: stepwise mutation model, TPM: two-phase model, Ho: The observed heterozygosity, Heq: The expected heterozygosity, Prob: The probability.

Table S10 Sign test and Wilcoxon signed-rank test to evaluate Jining Qing goat for mutation drift equilibrium under different models

| Population | Test method | Probability for one-tail test (heterozygosity excess) | | |
| --- | --- | --- | --- | --- |
|  |  | IAM | SMM | TPM |
| Yexianggu | Sign test | 0.379427 | 0.000132 | 0.014906 |
|  | Wilcoxon | 0.431641 | 0.000977 | 0.004883 |
| Mengman | Sign test | 0.324764 | 0.389583 | 0.602539 |
|  | Wilcoxon | 0.032227 | 0.695313 | 0.556641 |
| Kongge | Sign test | 0.482629 | 0.560196 | 0.516898 |
|  | Wilcoxon | 0.322266 | 0.695313 | 0.695313 |
| Mengla | Sign test | 0.395263 | 0.035372 | 0.542129 |
|  | Wilcoxon | 1.000000 | 0.128906 | 0.734375 |
| Menga | Sign test | 0.592396 | 0.172195 | 0.190935 |
|  | Wilcoxon | 0.375000 | 0.275391 | 0.845703 |
| Nangunhe | Sign test | 0.343439 | 0.175731 | 0.381746 |
|  | Wilcoxon | 0.375000 | 0.232422 | 0.845703 |

IAM: infinite alleles model, SMM: stepwise mutation model, TPM: two-phase model. *P* is the test of heterozygosity excess.
